# Bastardy Laws

**Published:** 1920-04-17

**Authors:** 


### Bastardy Laws.

### Terms of New Bill Published.

For some weeks, Mrs. Fisher (wife of the Education Minister) has been advocating with great persuasiveness a Bill dealing with the bastardy laws, mainly in the interests of the illegitimate child and also in favour of the mother. This Bill, which is presented by Mr. Neville Chamberlain, and backed by Lord Henry Bentinck, Mr. George Thorne, and Captain Loseby, has now been printed. Its general purpose is above reasonable controversy, and though there may be contentious clauses, every social reformer, and every lover of just dealing, must welcome its spirit and its effort to repair irregularities which have been allowed to exist far too long. The Bill is down for second reading on May 7, and it is much to be desired that the Government will give facilities for consideration of the measure so that it may in some form be incorporated in the law of the land. The Bill begins by requiring, on the registration of the birth of an illegitimate child, the filling up of a special form by the mother or other person responsible for the registration, in which the name of the alleged father of the child has to be stated, and whether he has admitted paternity, and, if so, to whom. The names of witnesses proving facts relating to the paternity are also asked for.

The Registrar of Births is to serve, within seven days afterwards, a notice on the alleged father, requiring him to state whether he admits paternity, and, if he does, what provision, if any, he is willing to make for the maintenance of the child, and whether he wishes to enter into a special agreement to be filed as an order of the Court. A reply must be made within eight days.

If the alleged father's offer is approved by two justices, it will be incorporated in an order. If it is not approved, or if the alleged father denies paternity, the clerk to the justices, or a collecting officer specially appointed, will take proceedings under the existing law. No compromise, dismissal by consent, or withdrawal of any proceedings shall be binding without the approval of the justices, after hearing the collecting officer.

A summons may be served on the alleged father before the birth of the child, and if he then admits paternity an order may be made forthwith, providing for weekly payments towards the support of the applicant until her confinement, and for its reasonable expenses. If the paternity in such a case is denied, the matter must be adjourned, but if after the birth the defendant is adjudged to be the putative father, the Court may order him to pay all the sums he would have been liable to in the first instance.

The maximum order which may be made against a father—formerly 5s. a week—is increased to 40s. in the Bill. If the mother has means and is not providing "suitable maintenance" for the child, she may be called upon by the collecting officer to do so, and, failing this, she, too, may be ordered by the justices to contribute a sum not exceeding 40s. a week in addition to any sums contributed by the father.

It is also proposed that proceedings may be instituted by a married woman in respect of an illegitimate child, and the marriage of the mother to another man is not to prevent the institution of affiliation proceedings against the alleged father of such a child.

Another provision is that the Registrar of Births shall notify the birth of every illegitimate child to the justices' clerk, and the mother or person having the care of such a child shall notify its full address within three months.

Every illegitimate child is to be a ward of the local Juvenile Court, and this Court may appoint a guardian for it, either in addition to its mother, or in substitution for her. Proceedings in these Courts are to be heard *in camera*.

The Bill legitimises children whose parents have afterwards married.

